# Supplementary material for: Identification of Key Genes Associated with Endoplasmic Reticulum Stress in Calcium Oxalate Kidney Stones
Source: Genes (Basel). 2025 Nov 6;16(11):1338. doi: 10.3390/genes16111338 (PMC12651965; doi:10.3390/genes16111338)
Supplement: Supplementary file 1 [file genes-16-01338-s001.zip › Supplementary Materials.pdf]

Supplementary Materials

Supplementary Figure S1 The pretreatment of single cell analysis. (A) Quality control of single cell data. **(B)** Top 2,000 highly variable genes. **(C)** Top 30 principle components (PCs) of single cell data. **(D)** Principal Component Analysis (PCA) plot of single-cell transcriptomes. The plot demonstrates the global transcriptomic profiles of Control and CaOx kidney stone (Case) samples, projected along the first two principal components (PC_1 and PC_2).

Supplementary Figure S2 Uniform manifold approximation and projection (UMAP) visualization of all single-cell transcriptomes from control (left) and CaOx kidney stone (right) samples. Each point represents an individual cell, colored by its annotated cell type.

Supplementary Table S1 Lists of 551 endoplasmic reticulum stress-related genes (ERSRGs) from GeneCards database with a relevance score ≥ 10

Supplementary Table S2 Lists of 3,483 key ERSRGs with |cor| > 0.1 and $P < 0.05$

Supplementary Table S3 Gene Ontology (GO) analysis of 3,483 key ERSRGs.

Supplementary Table S4 Kyoto Encyclopedia of Genes and Genomes (KEGG) enrichment analyses of 3,483 key ERSRGs based on Spearman analysis

Supplementary Table S5 The list of differentially expressed genes (DEGs) between high and low score groups

Supplementary Table S6 GO analysis of 851 DEGs

Supplementary Table S7 KEGG analysis of 851 DEGs

Supplementary Table S8 GO analysis of 9 key genes

Supplementary Table S9 KEGG analysis of 9 key genes
