# Supplementary figures and images for: Identification of Key Genes Associated with Endoplasmic Reticulum Stress in Calcium Oxalate Kidney Stones
Source: Genes (Basel). 2025 Nov 6;16(11):1338. doi: 10.3390/genes16111338 (PMC12651965; doi:10.3390/genes16111338)

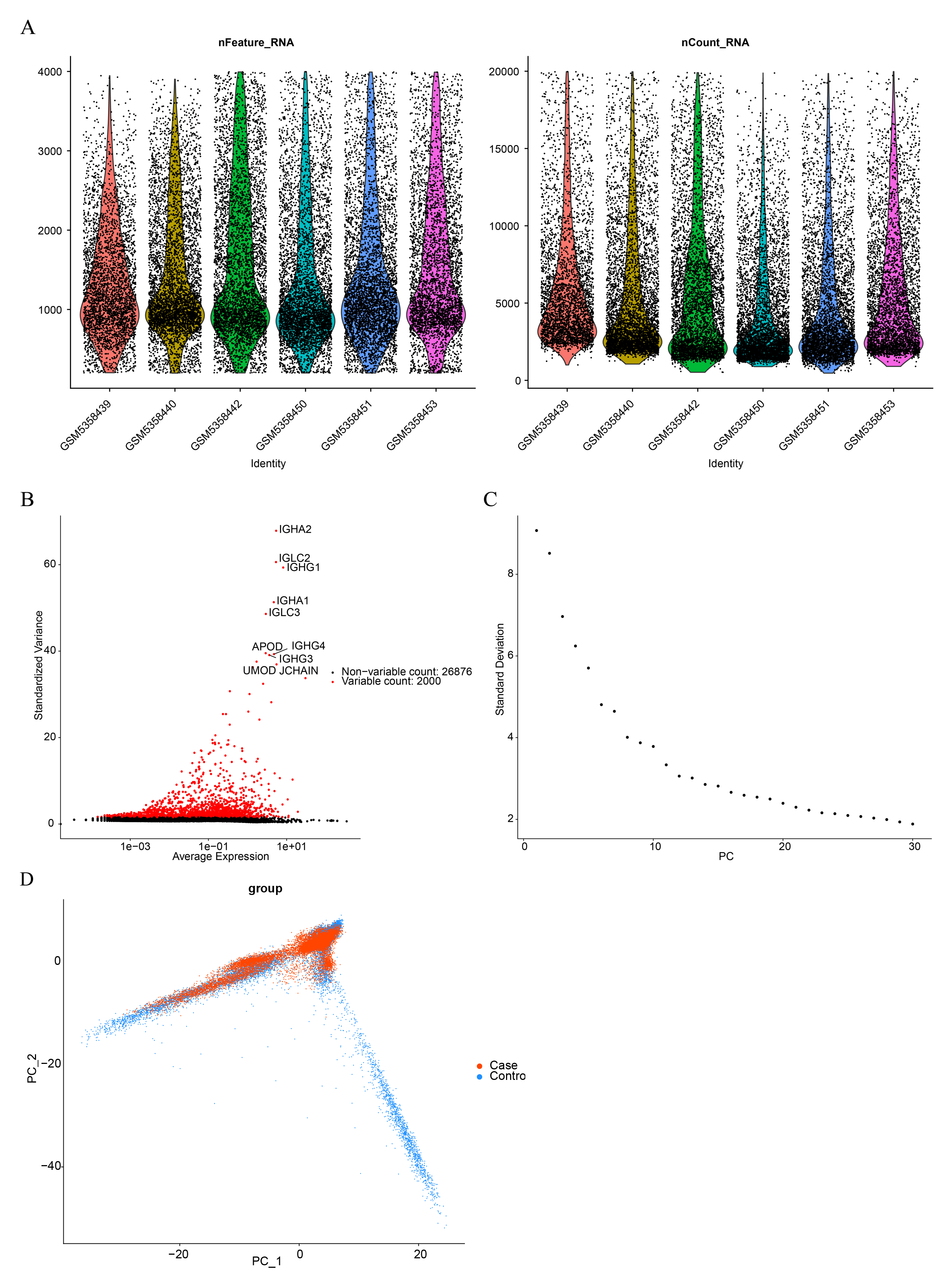

Supplement: Supplementary file 1 [file genes-16-01338-s001.zip › Supplementary Figure S1.tif]

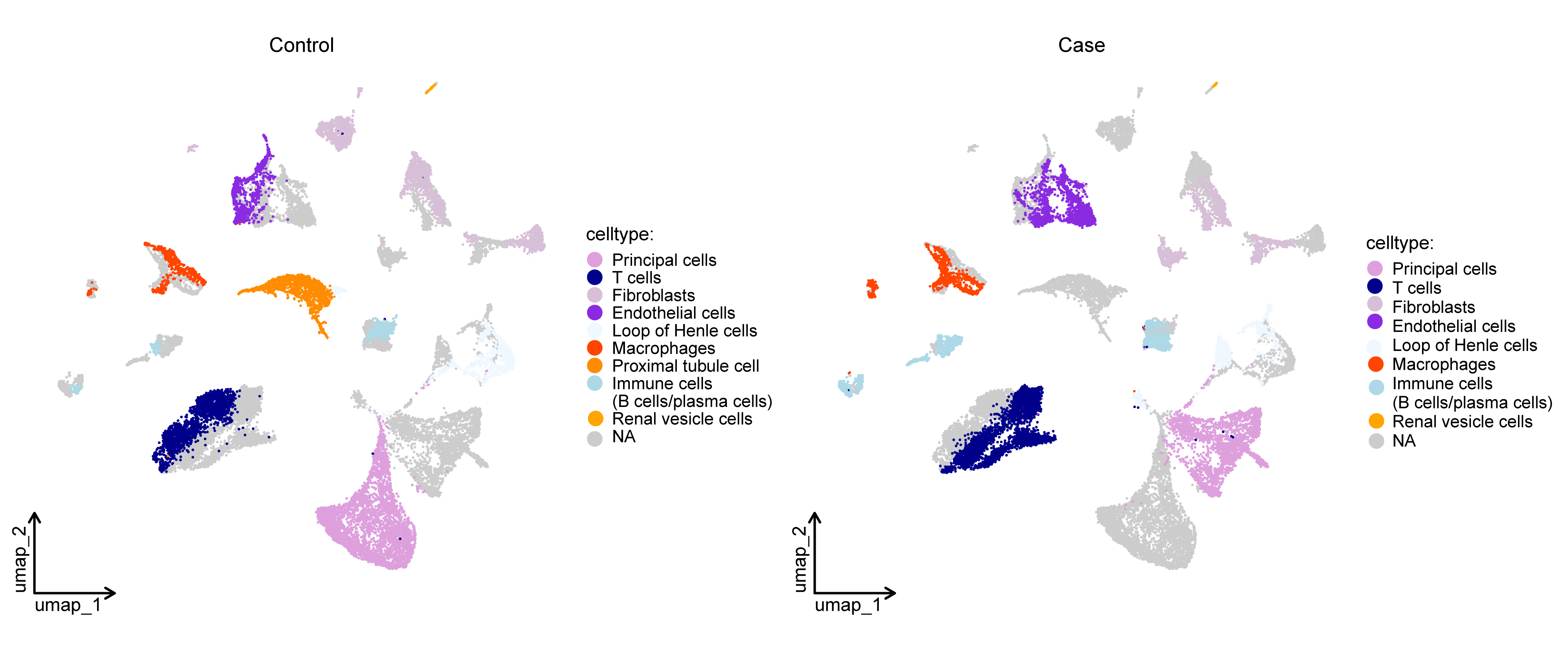

Supplement: Supplementary file 1 [file genes-16-01338-s001.zip › Supplementary Figure S2.tif]
